# Supplementary material for: Resolving the full spectrum of human genome variation using Linked-Reads
Source: Genome Res. 2019 Apr;29(4):635–45. doi: 10.1101/gr.234443.118 (PMC6442396; doi:10.1101/gr.234443.118)
Supplement: Supplemental Material [file supp_gr.234443.118_Supplemental_longranger-2.2.zip › longranger-2.2.tar.bz2!/lr2.2-public/lib/stan/cmdstan/stan/doc/api/html/namespacemembers_vars.html]

Stan: Namespace Members


|  |  |
| --- | --- |
|  | Stan  2.5.0  probability, sampling & optimization |


- Main Page
- Related Pages
- Namespaces
- Classes
- Files

- Namespace List
- Namespace Members

- All
- Functions
- Variables
- Typedefs
- Enumerations
- Enumerator

- a
- b
- c
- d
- e
- f
- i
- l
- m
- n
- p
- r
- s
- t
- u
- v


All Classes Namespaces Files Functions Variables Typedefs Enumerations Enumerator Friends Macros Pages

### - a -

- add\_conditional\_body\_f
  : stan::gm
- add\_conditional\_condition\_f
  : stan::gm
- add\_expression\_dimss\_f
  : stan::gm
- add\_fun\_var\_f
  : stan::gm
- add\_function\_signature\_f
  : stan::gm
- add\_loop\_identifier\_f
  : stan::gm
- add\_while\_body\_f
  : stan::gm
- add\_while\_condition\_f
  : stan::gm
- addition3\_f
  : stan::gm

### - b -

- binary\_op\_f
  : stan::gm

### - c -

- CONSTRAINT\_TOLERANCE
  : stan::math
  , stan::prob

### - d -

- data\_origin
  : stan::gm
- derived\_origin
  : stan::gm
- division\_f
  : stan::gm
- DOUBLE\_T
  : stan::gm

### - e -

- E
  : stan::math
- elt\_division\_f
  : stan::gm
- elt\_multiplication\_f
  : stan::gm
- EPSILON
  : stan::math
- exponentiation\_f
  : stan::gm
- expression\_as\_statement\_f
  : stan::gm

### - f -

- function\_argument\_origin
  : stan::gm
- function\_argument\_origin\_lp
  : stan::gm
- function\_argument\_origin\_rng
  : stan::gm

### - i -

- ILL\_FORMED\_T
  : stan::gm
- INFTY
  : stan::math
- INT\_T
  : stan::gm
- INV\_SQRT\_2
  : stan::math
- INV\_SQRT\_TWO\_PI
  : stan::math

### - l -

- left\_division\_f
  : stan::gm
- local\_origin
  : stan::gm
- LOG\_10
  : stan::math
- LOG\_2
  : stan::math
- LOG\_PI\_OVER\_FOUR
  : stan::math
- logical\_negate\_expr\_f
  : stan::gm

### - m -

- MAJOR\_VERSION
  : stan
- MATRIX\_T
  : stan::gm
- memalloc\_
  : stan::agrad
- MINOR\_VERSION
  : stan
- model\_name\_origin
  : stan::gm
- modulus\_f
  : stan::gm
- multiplication\_f
  : stan::gm

### - n -

- NEG\_TWO\_OVER\_SQRT\_PI
  : stan::math
- negate\_expr\_f
  : stan::gm
- NEGATIVE\_EPSILON
  : stan::math
- NEGATIVE\_INFTY
  : stan::math
- nested\_var\_alloc\_stack\_starts\_
  : stan::agrad
- nested\_var\_nochain\_stack\_sizes\_
  : stan::agrad
- nested\_var\_stack\_sizes\_
  : stan::agrad
- NOT\_A\_NUMBER
  : stan::math

### - p -

- parameter\_origin
  : stan::gm
- PATCH\_VERSION
  : stan

### - r -

- remove\_loop\_identifier\_f
  : stan::gm
- ROW\_VECTOR\_T
  : stan::gm

### - s -

- scope\_lp\_f
  : stan::gm
- set\_allows\_sampling\_origin\_f
  : stan::gm
- set\_fun\_type2\_f
  : stan::gm
- set\_fun\_type3\_f
  : stan::gm
- set\_fun\_type\_f
  : stan::gm
- set\_fun\_type\_named\_f
  : stan::gm
- set\_var\_type\_f
  : stan::gm
- set\_void\_function\_f
  : stan::gm
- SQRT\_2
  : stan::math
- subtraction3\_f
  : stan::gm

### - t -

- transformed\_data\_origin
  : stan::gm
- transformed\_parameter\_origin
  : stan::gm
- transpose\_f
  : stan::gm
- TWO\_OVER\_SQRT\_PI
  : stan::math

### - u -

- unscope\_locals\_f
  : stan::gm
- unscope\_variables\_f
  : stan::gm

### - v -

- validate\_allow\_sample\_f
  : stan::gm
- validate\_assignment\_f
  : stan::gm
- validate\_declarations\_f
  : stan::gm
- validate\_expr\_type3\_f
  : stan::gm
- validate\_int\_expr2\_f
  : stan::gm
- validate\_int\_expr3\_f
  : stan::gm
- validate\_integrate\_ode\_f
  : stan::gm
- validate\_non\_void\_arg\_f
  : stan::gm
- validate\_non\_void\_expression\_f
  : stan::gm
- validate\_return\_allowed\_f
  : stan::gm
- validate\_return\_type\_f
  : stan::gm
- validate\_sample\_f
  : stan::gm
- validate\_void\_return\_allowed\_f
  : stan::gm
- var\_alloc\_stack\_
  : stan::agrad
- var\_nochain\_stack\_
  : stan::agrad
- var\_stack\_
  : stan::agrad
- VECTOR\_T
  : stan::gm
- void\_function\_argument\_origin
  : stan::gm
- void\_function\_argument\_origin\_lp
  : stan::gm
- void\_function\_argument\_origin\_rng
  : stan::gm
- VOID\_T
  : stan::gm


---

[ Stan Home Page ]

*© 2011–2014,
Stan Development Team.*
